# Molecular detection and culture of *Mycobacterium avium* subsp. *paratuberculosis* in pasteurized and raw retail milk in Kampala, Uganda

**DOI:** 10.3389/fpubh.2026.1931886

**Published:** 2026-09-09

**Authors:** Judah Ssekitoleko, Auruga Christine Akello, Shariifah Kasinga, Magid Kisekka, Paula Emily Schweizer, Lonzy Ojok, Kamal H. Eltom, Uwe Truyen, Ahmed Abd El Wahed, Julius Boniface Okuni

**Affiliations:** 1 College of Veterinary Medicine, Animal Resources and Biosecurity, Makerere University, Kampala, Uganda; 2 Department of Livestock Health Research, Rwebitaba Zonal Agricultural Research and Development Institute, National Agricultural Research Organisation, Entebbe, Uganda; 3 Institute of Animal Hygiene and Veterinary Public Health, Leipzig University, Leipzig, Germany; 4 Department of Pathology, Faculty of Medicine, Gulu University, Gulu, Uganda; 5 Department of Animal Health and Safety of Animal Products, Institute for Studies and Promotion of Animal Exports, University of Khartoum, Shambat, Khartoum North, Sudan

**Keywords:** culture, milk, *Mycobacterium avium* subsp. *paratuberculosis*, public health, Uganda, zoonoses

## Abstract

**Background:**

The hypothesis that *Mycobacterium avium* subsp. *paratuberculosis* (MAP) is a zoonotic pathogen has gained traction with the repeated isolation of MAP from patients with Crohn’s disease. As consumption of contaminated milk and dairy products represents a primary suspected route for human exposure to MAP, this study was designed to investigate the presence of MAP in retail milk sold in Kampala, Uganda’s Capital and central hub for the nation’s milk distribution.

**Methods:**

A total of 772 milk samples were collected and analysed sequentially using two molecular assays targeting the *IS900* gene sequence, with positive findings validated by DNA sequencing. To determine MAP viability, samples were cultured on Herrold’s egg yolk media (HEYM) slants supplemented with selective antibiotics and mycobactin J.

**Results:**

MAP DNA was confirmed by nested PCR and subsequent sequencing in 2.3% of the samples, with equal frequencies in pasteurized and unpasteurized milk. Additionally, one raw milk sample demonstrated viable MAP growth by culture.

**Conclusion:**

The findings of contamination of commercial milk with MAP in the Uganda’s Capital aligns with the reported prevalence of cattle paratuberculosis in the country and underscore the need for rigorous protocols for preparation of milk and dairy products to reduce contaminating viable pathogenic bacteria including MAP. Consequently, more efforts toward control of paratuberculosis in livestock are required to mitigate possible public health risks through foodborne pathogens.

## Introduction

*Mycobacterium avium* subsp. *paratuberculosis* (MAP) is the known cause of paratuberculosis in cattle and other ruminant animals (1) and a suspected etiology of Crohn’s disease (2–5) and many other chronic diseases in humans (6). Although no conclusive evidence has been provided yet, the persistent isolation of MAP in patients with Crohn’s disease and related conditions has fuelled the suspicion that the bacterium plays a crucial role in the pathogenesis of these diseases (4, 7) placing it as a potential zoonotic pathogen.

It has been postulated that humans get infected with MAP when they consume animal products such as milk and meat that have been contaminated with the organism (8). In several communities, animals have a close connection with humans and, in many instances, people consume raw or poorly prepared animal products such as milk and meat (9).

The continued detection of MAP in milk and other animal products presents a big challenge (10, 11). However, the presence of MAP DNA in these products does not necessarily confirm that the bacteria are alive, though some studies have reported occurrence of viable MAP in both raw and pasteurized milk by culture, or by phage amplification methods which only target viable bacteria (12, 13). MAP detection in milk has been documented globally. Studies have detected viable MAP in retail pasteurized and unpasteurized milk across various countries, including the United States of America, where 2.8% of viable MAP was detected in retail pasteurized milk (14), India (10), Chile (15), Italy (16) and Ireland (17) and Uganda in pooled raw milk samples (18).

Although MAP prevalence has been documented in local cattle herds in Uganda (19, 20) in addition to the above- mentioned report in raw milk made by Okuni and others, no further studies have investigated presence of the viable MAP in retail unpasteurized or pasteurized milk, taking in consideration the zoonotic potential of this organism.

Therefore, the current study aimed at investigating the extent of contamination of cattle milk with MAP in Kampala district of Uganda, which could as well reflect the situation in other parts of the country.

## Materials and methods

### Study area and sample collection

**Figure 1:**
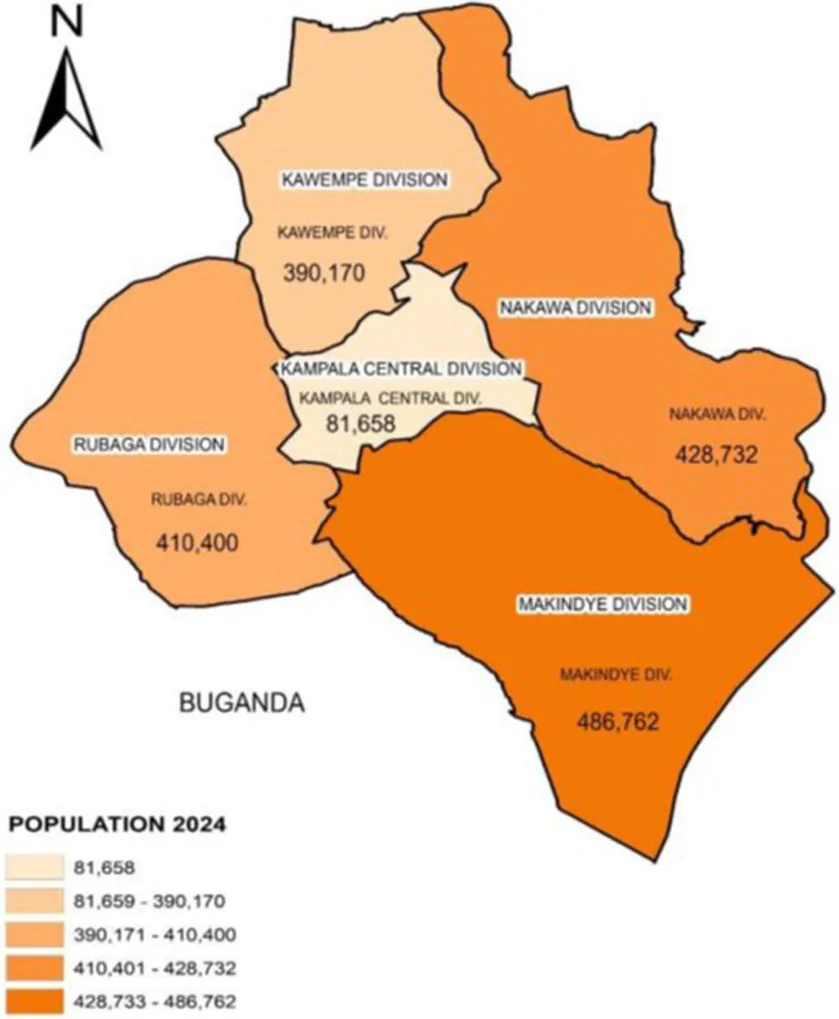
Map of Kampala Capital City showing the 5 divisions and the human population distribution. Source: www.ubos.org/nphc-2024.

Raw/unpasteurized and pasteurized milk samples were collected between January 2025 and October 2025 from randomly selected milk outlets across the 5 divisions of Kampala district (Uganda), namely: Central, Rubaga, Kawempe, Nakawa and Makindye (Figure 1). The samples were purchased from shops selling pasteurized milk (two main brands) and non- pasteurized milk in the five divisions of Kampala.

The WINPEPI^®^ program for epidemiologists (21) (version 11.65) was used to estimate the sample size. The calculation assumed a design effect of two, acceptable difference of 2% and an assumed proportion of 4.2% (18) at 95% confidence level. A multi-stage random sampling was employed in which five wards (cells) were selected randomly from a division. From each of the five wards, all shops vending milk were sampled in all the five divisions in the district. Thus, the total number of milk samples collected in the district (N), was a sum of all samples from each of the five divisions (n), calculated as, N = n_1_ + n_2_ + n_3_ + n_4_ + n_5_. All samples were purchased in their original retail packaging of 500 mL and then transported to the laboratory in ice-cooled boxes within 2 h of purchase and stored at 4 °C until processing.

### DNA extraction

**Figure 2:**
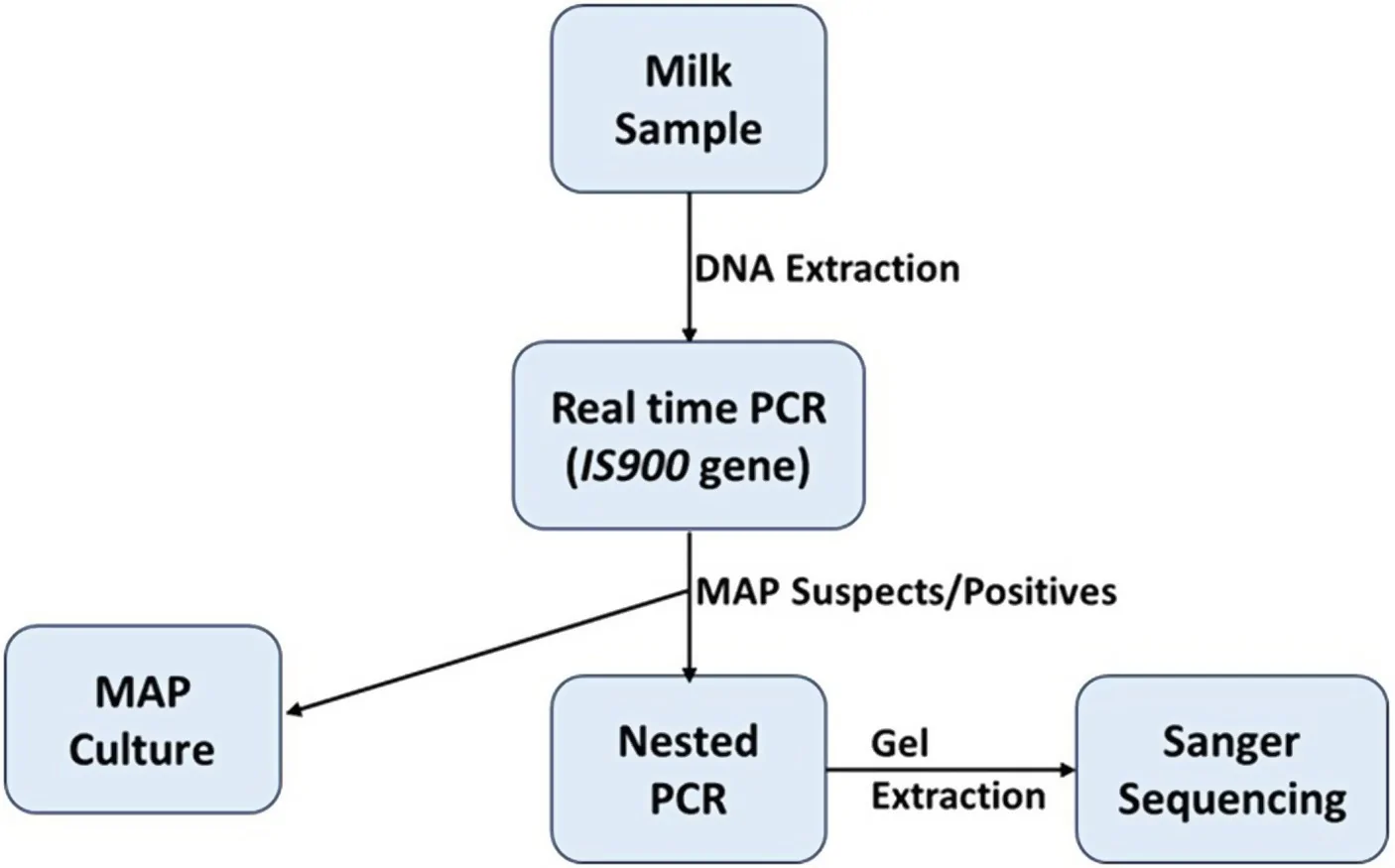
Illustration of the tests done for the detection of *Mycobacterium avium subsp. paratuberculosis* in milk samples.

From each milk sample, 2 mL was aliquoted into 2-ml Eppendorf tube (Eppendorf, Hamburg Germany) and centrifuged at 12,000× g for 5 min. The supernatant was carefully discarded while retaining the fat layer and the pellet for DNA extraction. Genomic DNA was extracted using the GeneJET Genomic DNA Purification Kit (Thermo Fisher Scientific Baltics UAB, Vilnius, Lithuania) according to the manufacturer’s instructions with the following critical modification to improve lysis of the recalcitrant mycobacterial cell wall: 180 μL of mycobacterial lysis buffer (10% SDS, 1M EDTA, 1M Tris–HCl, 1M NaCl and 1.2% Triton X) was added to the pellet and incubated at 80 °C for 15 min with occasional vortexing, followed by proteinase K and RNase treatment, respectively. Following a series of steps involving incubation, washing, vortexing and centrifugation, the DNA was finally eluted in 50 μL of elution buffer and quantified using a Qubit 4 Fluorometer (Invitrogen by ThermoFisher Scientific, Life Technologies Holdings Pte Ltd., Singapore). Extracted DNA was stored at −20 °C until use. An illustration of the subsequent tests and analyses is given in Figure 2.

### Real-time PCR screening for MAP

Extracted DNA was tested for the presence of MAP DNA using a real-time PCR assay targeting the *IS900* insertion sequence with primers and probes as follows; MAP-PCR-FP = 5′-TAC CGC GGC GAA GGC AAG AC-3′, MAP-PCR-RP = 5^′^-CGG AAC GTC GGC TGG TCA GG-3′, MAP-PCR-Probe = 5′-[FAM] A TGA CAT CGC AGT CGA GCT G [HQ1]-3′ (22). The reaction total volume was 20 μL consisting of 10.0 μL of LightCycler 480 Probes Master, 0.5 μL of each of the forward and reverse primer (10 μM), 1.0 μL of probe (10 μM), 3.0 μL of molecular grade water and 5.0 μL of DNA template on a LightCycler 480 (Roche Diagnostics) real-time PCR system. The temperature profile included an initial denaturation at 95 °C for 10 min followed by 45 cycles comprising denaturation at 95 °C for 15 s, annealing at 60 °C for 30 s and extension at 72 °C for 35 s according to the manufacturer’s instructions. Each run included a positive control of MAP-K10 strain, which is well characterized, and a negative control of molecular grade water. Samples with a cycle threshold (Ct) value ≤ 35 and a characteristic amplification curve were considered positive whereas samples with a Ct value between 35 and 40 were considered suspect and were all proceeded to the nested PCR.

### Nested PCR for MAP confirmation

All real-time PCR-positive or suspect samples were subjected to nested PCR for additional sensitivity. The first PCR round amplified a larger fragment of the *IS900* region of 413 bp using outer primers with the following sequences, Forward primer Map1F = 5´-CATCGGAACGTCGGCTGGTCAGG-3′, Reverse primer Map1R = 5´-GATCGCCTTGCTCATGGCTGCCG-3′ (23). The total reaction mix was 12.5 μL: 1.0 μL of 10 mM of each primer, 6.25 μL of standard Master Mix (Taq 2x Master Mix, New England BioLabs Inc), 3.25 μL of molecular grade water and 1.0 μL of DNA template. The thermal conditions used were initial denaturation of 95 °C for 3 min., followed by 35 cycles with denaturation at 95 °C for 30 s, annealing at 63 °C for 30 s, extension at 68 °C for 60 s and final extension at 68 °C for 5 min. The second PCR round targeted an inner region within the amplified 413 bp—fragment using the following primers: Forward primer Map2F = 5´-GCAGCTCGACTGCGATGTCATCG-3′, Reverse primer Map2R = 5´-GGCACGGGCTGCTTTATATTCCC-3′. The reaction mix was 20 μL consisting of 1.0 μL of 10 mM of each primer, 10.0 μL of standard Master Mix (Taq 2x Master Mix), 6.0 μL of molecular grade water and 2.0 μL of the PCR product of the first round as DNA template. The thermal conditions used were initial denaturation of 95 °C for 3 min and then 35 cycles with denaturation at 95 °C for 30 s, annealing at 60 °C for 30 s, extension at 68 °C for 60 s and final extension 68 °C for 5 min to generate an approximately 230 bp amplicon. The PCR products were then visualized by electrophoresis on 1.5% agarose gels stained with ethidium bromide under UV transillumination; a DNA ladder of 100 bp was included (New England, Biolabs, 100–1,517 bp, Cat. no. N3231L). A clear visible band of about 230 bp was considered positive.

### Gel extraction and sanger sequencing

Nested PCR amplicons from all confirmed positive samples were excised from the agarose gel and purified using the QIAquick Gel Extraction Kit (QIAGEN, Hilden, Germany) following the manufacturer’s protocol. Purified amplicons were quantified and sent to Inqaba Biotech Africa Genomics (South Africa) for Sanger sequencing using the inner primers. Sequences were edited using FinchTV software, then subjected to BLASTn analysis against the NCBI GenBank nucleotide database.

### Milk culture

All MAP positive and suspect samples were cultured to determine MAP viability. For this, 40 mL of each PCR- positive or suspected milk sample was centrifuged at 2,500× *g* for 15 min to obtain a pellet. The pellet and cream layer were resuspended in 10 mL of 0.75% hexadecylpyridinium chloride (HPC), followed by incubation at room temperature for 5 h, then centrifuged at 2,500× *g* for 15 min. The resulting pellet was resuspended in 1 mL of PBS followed by inoculation onto Herold’s egg yolk medium (HEYM) slants supplemented with mycobactin J and antibiotics (Vancomycin, Amphotericin B and Nalidixic acid). Inoculated slants were incubated at 37 °C for 12–24 weeks, with weekly examination for positive growth. Suspected colonies were tested for acid-fastness by Ziehl-Neelsen staining and confirmed as MAP by the *IS900* PCR as described earlier. Negative culture results were reported after 24 weeks of incubation with no growth.

### Quality control and data analysis

All DNA extractions and PCR runs included extraction blanks and no-template controls to monitor contamination. Positive real-time PCR results were only accepted after confirming the sequence identity of the nested PCR amplicons. All data were compiled in Microsoft^®^ Excel sheet and positivity rates were calculated as the proportion of confirmed positive samples out of the total examined, stratified by pasteurization status. To identify significant differences (*p* < 0.05), among the distribution values within the division strata, Chi-square and Fisher’s exact tests were performed using *R* (24).

## Results

### Detection and distribution of MAP in Kampala’s milk supply

In the current study, we investigated MAP contamination of milk sold in Kampala district. As Uganda’s central business hub, Kampala receives milk from various regions nationwide. A total of 772 samples of both pasteurized and unpasteurized milk were collected from the 5 divisions of Kampala district.

**Figure 3:**
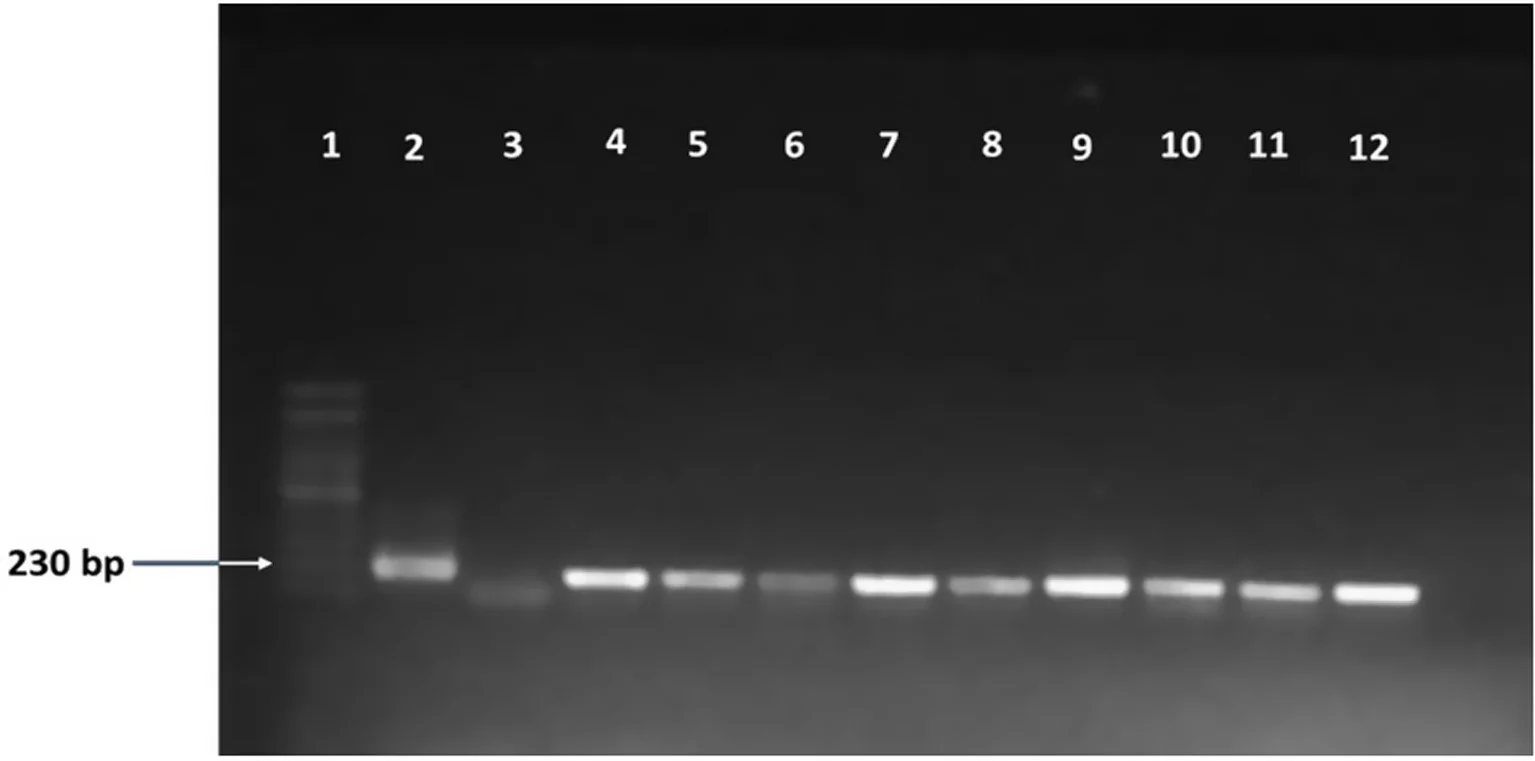
Representative agarose gel electrophoresis results of 9 milk samples tested for MAP DNA by nested PCR. Lane 1: 100 bp ladder; Lane 2: Positive control; Lane 3: Negative control; Lanes 4–12: Samples (U313, U465, U363, U388, U273, P153, P025, P030, U028), where U and P stand for unpasteurized and pasteurized, respectively.

MAP detection was done by real-time PCR followed by nested PCR for MAP confirmation. Out of the 772 milk samples, 26 (3.36%) gave a weak MAP positive signal (Ct 35–40), whereas 2 samples gave a definitive MAP positive signal via the real-time PCR assay (Supplementary file 1). Of the 28 weakly- positive milk samples, 18 were confirmed positive by nested PCR, while the remaining 10 tested negative. For this, a representative gel of nine MAP-positive milk samples is given in Figure 3. Overall, the confirmed MAP-positive samples accounted for 2.3% of the total number of samples. The detailed breakdown of these findings across the study area is presented in Table 1.

**Table 1:** Prevalence of *Mycobacterium avium* subsp. *paratuberculosis* across the 5 divisions of Kampala district.

Division	Pasteurized	Raw	Total samples	MAP+ (nested PCR)		MAP positivity rate (%) (95% CI)
				Pasteurized	Raw	
Kawempe	140	164	304	4 (2.9%)	5 (3%)	2.96 (1.57–5.53)
Makindye	58	120	178	1 (1.7%)	0 (0%)	0.56 (0.10–3.11)
Nakawa	44	112	156	2 (4.5%)	3 (2.7%)	3.2 (1.38–7.28)
Rubaga	55	47	102	1 (1.8%)	1 (2.1%)	1.96 (0.54–6.87)
Central	24	8	32	1 (4.1%)	0 (0%)	3.12 (0.55–15.74)
Total	**321**	**451**	**772**	**9 (2.8%)**	**9 (2%)**	**2.3% (1.48–3.66)**

Positivity rates are presented with 95% confidence intervals (CI).

To determine whether significant differences existed among the pasteurized and raw milk sample values, a Pearson’s chi-square test was used; however, no significant differences were revealed (*p-value* = 0.22). Among the five divisions, Nakawa registered the highest positivity rate (3.20%), followed by Central division (3.12%) and Kawempe at 2.96% (Table 1). However, there was no significant difference in frequencies between the pasteurized and raw milk groups (Fisher’s exact test, *p-value* = 0.48).

### MAP culture and sequence analysis

Out of the 28 cultured milk samples, only one unpasteurized sample (U465) produced acid-fast colonies and tested positive for the MAP *IS900* gene via real-time PCR. Of the 28 samples, 13 were pasteurized while 15 were unpasteurised. Sequence analysis of the nested PCR products revealed 100% identity with the *IS900* gene of the MAP_K10 reference strain.

## Discussion

There is a growing suspicion that MAP is a zoonotic pathogen driven by its consistent isolation from patients with Crohn’s disease and other chronic inflammatory bowel diseases (25, 26, 6). It is hypothesized that MAP transmission to humans occurs through consumption of milk and other animal products contaminated with MAP. Supporting this link, several studies have isolated MAP in both pasteurized and unpasteurized milk, highlighting commercial dairy as a potential source of infection for humans (27, 13). However, the data situation on the epidemiology of MAP in humans remains scarce, especially in developing countries (28).

There are several communities in Africa where milk is frequently consumed raw or undergoes insufficiently processing prior to consumption (29, 30). Furthermore, commercial milk processing is limited by deficient power supplies and lack of the necessary equipment for milk processing; consequently, dairy products are produced under suboptimal hygienic conditions (31, 32). These deficiencies inadvertently expose unsuspecting consumers to foodborne microbial pathogens, including MAP.

Several approaches are used to lower the presence of harmful bacteria including MAP in dairy products which are intended for human consumption. The commonly used method is pasteurization which can be carried out using different techniques such as high-heat short-time (72 °C for 15 s) or low-heat long-time (63 °C for 30 min) and ultra-pasteurization or ultra-high temperature (33, 34). Pasteurization effectiveness is highly influenced by the duration and temperature applied; the high-temperature short-time method of about 72 °C for 15 s has been generally considered efficient in reducing MAP levels (35). However, complete inactivation is not guaranteed, it largely depends on the initial bacterial load as well as the pasteurization conditions (36).

In the current study, we investigated MAP contamination of milk sold in Kampala district. As Uganda’s central business hub, Kampala receives milk from various regions nationwide. Among its five divisions, Kawempe registered the highest density of milk sales points, driven by its large residential population relative to the commercially concentrated Central division. MAP DNA was detected in 2.3% of the samples in both pasteurized and unpasteurized milk. However, significance level tests revealed no differences (*p-value* > 0.05) in the milk sample distribution values and in the positivity rates among the pasteurized and raw milk sample strata. Sequencing of the amplified target DNA band confirmed presence MAP according to the IS900 insertion sequence which is highly conserved in the MAP genome (37).

While MAP DNA was detected at equal frequencies in the pasteurized and unpasteurized milk, the pasteurized milk registered a higher positivity rate. It is worth noting that most of the PCR positive samples could be resulting from non-viable DNA, though some is likely to have originated from viable bacteria, consistent with previous reports of viable MAP in pasteurized retail milk (14, 27, 11). However, culture attempts in the current study yielded one positive unpasteurized sample (U465); confirmed via IS900 real-time PCR. This sample Ct value was in the same range as the other samples which did not grow. The limited culture growth therefore observed may be attributed to low initial bacterial loads, a slow growth rate, as well as the fastidious nature of the organism. Given the low sensitivity of conventional culture methods, future investigations should consider to incorporate alternative techniques, such as phage-based assays, to enhance the detection of viable cells.

The study highlights the critical need for community-level awareness campaigns on the importance of proper milk hygiene practices to curb possible harmful effects due to unsafe milk consumption. However, some limitations to the study were observed: the investigation did not evaluate MAP in other dairy products such as yoghurt or cheese which could as well pose alternative routes for exposure. Additionally, future research should investigate other parts of the country especially milk collection hubs in urban centres in the region. The broader geographic sampling will enable assessment of the national milk hygiene landscape and inform targets for intervention. Because milk and its products are widely consumed often with minimal processing, the findings of this research carry significant implications for food safety.

## Conclusion

This study has confirmed the presence of MAP in retail milk in Kampala district and it aligns with previous research that reported considerable MAP prevalence in local cattle herds. MAP was detected in 18 of the 772 milk samples tested and in equal proportions between the pasteurized and the raw retail milk. The study has also demonstrated that some of the observed MAP DNA in milk is viable with potential to persist in human food if adequate precautionary measures are not taken. The widespread contamination of human food supplies—coupled with growing evidence of MAP’s zoonotic potential—presents a significant public health concern. Consequently, implementing strict MAP control measures in livestock and adherence to dairy processing protocols is urgent to mitigate the risk of foodborne human infections.

## Data Availability

The datasets presented in this study can be found in online repositories. The names of the repository/repositories and accession number(s) can be found in the article/Supplementary material.
