# Supplementary material for: Molecular detection and culture of Mycobacterium avium subsp. paratuberculosis in pasteurized and raw retail milk in Kampala, Uganda
Source: Front Public Health. 2026 Sep 9;14:1931886. doi: 10.3389/fpubh.2026.1931886 (PMC13597795; doi:10.3389/fpubh.2026.1931886)
Supplement: Supplementary file 1 [file Supplementary_file_1.docx]

**Supplementary file 1: List of suspect and MAP positive milk samples tested on real-time PCR followed by nested PCR**

| **S. No** | **Lab ID** | **Division** | **Real time PCR**  **(Ct value)** | **Nested PCR**  **(Gel electrophoresis)** | |
| --- | --- | --- | --- | --- | --- |
|  |  |  |  | **Positive** | **Negative** |
| 1 | P025 | Kawempe | 38.51 | + |  |
| 2 | P018 | Kawempe | 39.59 | + |  |
| 3 | P058 | Rubaga | 37.57 |  | - |
| 4 | P020 | Kawempe | 37.35 | + |  |
| 5 | P027 | Kawempe | 34.63 | + |  |
| 6 | U060 | Makindye | 37.83 |  | - |
| 7 | P100 | Nakawa | 37.54 |  | - |
| 8 | U164 | Kawempe | 37.61 | + |  |
| 9 | P124 | Nakawa | 38.98 | + |  |
| 10 | U463 | Nakawa | 37.76 | + |  |
| 11 | P153 | Central | 36.24 | + |  |
| 12 | U028 | Rubaga | 34.79 | + |  |
| 13 | U009 | Kawempe | 38.09 | + |  |
| 14 | U079 | Makindye | 38.94 |  | - |
| 15 | P084 | Makindye | 36.92 |  | - |
| 16 | U313 | Kawempe | 36.20 | + |  |
| 17 | U465 | Nakawa | 37.30 | + |  |
| 18 | U363 | Kawempe | 36.32 | + |  |
| 19 | U026 | Rubaga | 37.61 |  | - |
| 20 | P030 | Rubaga | 39.45 | + |  |
| 21 | P073 | Makindye | 36.56 | + |  |
| 22 | P113 | Nakawa | 35.03 | + |  |
| 23 | P051 | Rubaga | 37.65 |  | - |
| 24 | U075 | Makindye | 38.48 |  | - |
| 25 | U092 | Makindye | 37.71 |  | - |
| 26 | U110 | Nakawa | 38.22 |  | - |
| 27 | U388 | Kawempe | 38.21 | + |  |
| 28 | U273 | Nakawa | 37.43 | + |  |
|  | **Total** |  |  | **18** | **10** |
